# Evaluation of the necessity of simultaneous cholecystectomy in patients undergoing liver hydatid cyst surgery

**DOI:** 10.1016/j.sopen.2025.02.009

**Published:** 2025-02-26

**Authors:** Hüseyin Fahri Martlı, Arzu Hazal Aydın, Eda Şahingöz, Derviş Duru, Sadettin Er, Nesrin Turhan, Mesut Tez

**Affiliations:** a Ankara Bilkent City Hospital, General Surgery Department Üniversiteler Mah. Dumlupınar Cad, Çankaya, Ankara, Turkey; b Department of Pathology, Aksaray University Aksaray Training and Research Hospital, Aksaray 68200, Turkey; c Ankara City Hospital, Pathology Department Üniversiteler Mah. Dumlupınar Cad, Çankaya, Ankara, Turkey

**Keywords:** Chyst hydatid, Chystectomy, Cholelitiazis, Cholecystectomy

## Abstract

**Introduction:**

Liver hydatid cysts remain a significant public health issue in Turkey, the Middle East, East Asia, and Russia. Surgical treatments are often employed for certain stages of this disease. However, the necessity of simultaneous cholecystectomy during these procedures remains unclear. Treating symptoms related to subsequent cholelithiasis can become more challenging. This study investigates the necessity of simultaneous cholecystectomy by following patients who underwent hydatid cyst surgery with or without concurrent cholecystectomy.

**Materials and methods:**

Patients who underwent surgery for hydatid cysts between 2019 and 2024 at the General Surgery Clinic of Ankara Bilkent City Hospital were retrospectively reviewed. A total of 97 patients were included, with 56 (54.32 %) undergoing cholecystectomy along with hydatid cyst surgery (Group 1) and 41 (45.68 %) not undergoing cholecystectomy (Group 2).

Preoperative clinical, laboratory, and radiological findings, as well as intraoperative data, morbidity, mortality, and postoperative symptoms, were analyzed.

**Results:**

Patients in Group 1 had longer hospital stays, higher blood loss, and significantly higher Clavien-Dindo complication scores. In the postoperative follow-up of Group 2, 8 patients (19.51 %) developed stones or sludge, and 1 patient (2.4 %) developed polyps. Four patients (9.75 %) presented to the emergency department with cholecystitis symptoms. A total of 5 patients (12.19 %), including 4 with symptomatic cholelithiasis (9.7 %) and 1 with gallbladder polyps (2.4 %), underwent elective cholecystectomy. Two (40 %) of these cholecystectomies were performed laparoscopically, while three (60 %) were converted to open cholecystectomy.

**Conclusion:**

Simultaneous cholecystectomy during liver hydatid cyst surgery may prevent difficulties associated with treating symptoms related to subsequent cholelithiasis.

## Introduction

Liver hydatid cysts are an endemic parasitic disease in countries where rural living is prevalent, such as Eastern Europe, Mediterranean countries, East Asia, the Middle East, China, India, Russia, and South America [1]. Hydatid cysts are transmitted to humans by oral ingestion of cyst eggs via food contaminated with the feces of infected dogs, and the organ most commonly affected is the liver [1,2]. Proper radiological classification of liver hydatid disease is crucial for appropriate treatment planning). According to the World Health Organization (WHO) classification, Type 1 cysts are unilocular and purely cystic; Type 2 cysts have a multilocular, honeycomb-like appearance; Type 3 cysts contain daughter vesicles that have separated from the germinative membrane or are embedded in a solid matrix; Type 4 cysts lack daughter vesicles and may have partial calcifications; and Type 5 cysts have a solid and calcified cyst wall. Surgical treatments are recommended for WHO Type 3 (Gharbi Type 2) hydatid cysts, cysts that have perforated, or those opening into the bile ducts [1,3,4].

Hydatid cyst surgery can be classified as conservative (e.g., cyst aspiration, cystotomy, capitonnage) or radical (e.g., segmentectomy, lobectomy, pericystectomy) approaches [[5], [6], [7]].

Additionally, for hydatid cysts opening into the bile ducts, T-tube drainage may be added to the primary procedure after bile duct exploration [7,8]. Cholecystectomy is indicated in cases where the hydatid cyst extends to the gallbladder or in rare cases of primary gallbladder hydatid cyst [9].

Although there are publications suggesting that the gallbladder can be preserved during major hepatectomy surgeries, cholecystectomy is traditionally recommended [10,11]. This recommendation is based on the potential risk of cholelithiasis due to disruption of the gallbladder's neural innervation and anatomy during liver surgery [12]. However, the necessity of performing cholecystectomy during conservative hydatid cyst surgery has not been widely discussed in the literature. While some studies report cholecystectomies performed due to biliary tract exploration or hydatid cysts extending into the gallbladder, prophylactic cholecystectomy is not commonly addressed [5,6,9,[13], [14], [15]]. In this study, we aimed to investigate whether simultaneous cholecystectomy is necessary during hydatid cyst surgery by analyzing the pathology results of patients who underwent cholecystectomy and the clinical follow-up outcomes of patients who did not.

## Materials and methods

### Patients and methods

Patients who were followed for hydatid cysts at Ankara Bilkent City Hospital between March 2019 and March 2024 were retrospectively reviewed. A total of 97 patients who underwent surgery due to WHO type 3 classification for active hydatid cysts that had opened into the bile ducts, hydatid cysts causing pressure symptoms and pain (of any type), any type that did not benefit from non-operative interventions, or perforated hydatid cysts were included in the study. Of these, 56 (54.32 %) underwent cholecystectomy along with hydatid cyst surgery (Group 1), while 41 (45.68 %) were followed without cholecystectomy (Group 2).

At our center, a routine cystic duct leak test is performed on patients with hydatid cysts that have opened into the bile ducts. For that reason, cholecystectomy is performed on all patients with bile leakage from the cyst cavity; these patients were included in Group 1.

Demographic characteristics such as age, sex, contact with dogs, and history of hydatid cysts; clinical features such as presenting complaints and physical examination findings; laboratory data including hemoglobin levels, eosinophil count, eosinophil percentage, liver function tests, kidney function tests, cholestasis markers, and hydatid cyst indirect hemagglutination test results; radiological investigations such as ultrasound, computed tomography (CT), and magnetic resonance imaging (MRI); and radiological findings such as the type of hydatid cyst, bile duct involvement, compression on vascular, biliary systems, and other structures; as well as the cyst wall pathology report were all obtained from electronic medical records.

The pathology reports of cholecystectomy specimens from Group 1 patients were re- examined by pathologists. Group 2 patients' follow-up comprised an assessment of hospital admission complaints, laboratory and radiographic testing, and whether they were diagnosed with cholelithiasis and had cholecystectomy.

Patients under 18 years of age, those diagnosed with hydatid cysts treated with medical therapy or percutaneous aspiration inspiration reaspiration (PAIR) procedures, patients with WHO type 4 or 5 hydatid cysts without treatment indication (except those with compression symptoms), and patients who underwent surgery for *Echinococcus multilocularis* were excluded from the study.

Approval for this study was obtained from the Ethics Committee of Ankara Bilkent City Hospital with the approval number E-244514810. The article was prepared following STROBE guidelines.

### Statistics

Data were analyzed using descriptive statistics to summarize the clinical characteristics, demographic variables, and outcomes of the patients. Continuous variables are expressed as mean ± standard deviation (SD) or median with interquartile ranges (IQR) depending on the distribution of the data, which were assessed using the Shapiro–Wilk test.

Categorical variables are presented as frequencies and percentages. Comparisons between groups were performed using the chi-square test or Fisher's exact test for categorical variables, and the Student's *t*-test or Mann-Whitney *U* test for continuous variables, depending on the normality of the data distribution. Statistical significance was set at a p- value of <0.05. All statistical analyses were performed using SPSS version 26.0 (IBM Corp., Armonk, NY, USA).

## Results

The median age of the 97 patients included in the study was 43.6 years (min/max: 020/079); 045 (46.3 %) were female, and 052 (53.6 %) were male. In Group 2, no gallstones were detected in the preoperative ultrasound examinations. In Group 1, preoperative imaging revealed gallstones or sludge in 12 patients (21.4 %), while 44 patients (78.6 %) had no gallstones or sludge. None of the patients with gallstones or sludge in their gallbladders exhibited any symptoms related to the gallbladder (Table 1).

**Table 1:** Demographics, clinics, radiologics data of patients.

	Group 1(N:56)	Group 2(N:41)	p
	N (%)(min -max)	N(%) (min-maks)	
Age	47(20–79)	39(20–71)	0.078
Gender			0.055
Male	25(%44.6)	27 (65.9 %)	
Female	31(%55.4)	14(34.1 %)	
Cholelytiazis			
No	41(%73.2)	41(100 %)	0.0658
Yes	14(%25)	0	–
Gallbladder Polyp			
No	55 (%98.2)	41(100 %)	0.0534
Yes	1(%1.8)	0	
Hydatide Cyst Type (WHO Classification)			
Type 1	5(%8.9)	1(2.43 %)	0.0836
Type 2	14(%25)	4(9.75 %)	**0.023**
Type 3	33(%58.9)	36(87.8 %)	**0.048**
Type 4	3(%5.4)	0	–
Type 5	1(%1.8)	0	–
Liver Localisation of Cyst			
Single	13(%23.2)	29(70.73 %)	**0.02**
Multiple	43(%76.8)	12(29.27 %)	**0.021**
Cyst Size	8.5 cm(4-20 cm)	6.2 cm(4-14 cm)	0.065
Numune Score	3,24(1–4)	1.73(1–4)	**0.002**
Operation Procedure			
Conservative	52(%92.9)	41(100 %)	0.077
Radicale	4(%7.14)	0	–
Operation Type			
Laparascopicaly	12(21.42 %)	16(39.02 %)	**0.005**
Open	44(78.57 %)	25(60.08 %)	**0.048**
Operation Emergency			
Emergency	4(7.14 %)	1(2.43 %)	**0.038**
Elective	52(92.86 %)	40(97.57 %)	0.211

When evaluating the pathology results of patients in Group 1, 050 (89.2 %) were diagnosed as having chronic cholecystitis. Among these, 14 (28 %) had accompanying gallstones or sludge, and 1 (2 %) had a polyp. Additionally, 5 (10 %) of these patients had eosinophilic infiltration along with chronic cholecystitis; of these, 3 (60 %) had gallstones, while 2 (40 %) did not. The pathology reports of the 6 patients (10.7 %) who did not have a diagnosis of chronic cholecystitis described the gallbladder material as showing no significant pathology. In Group 1, the hydatid cysts of 35 patients (62.5 %) had opened into the bile ducts. Furthermore, all patients with eosinophilic infiltration in the gallbladder had hydatid cysts that opened into the bile ducts (Table 2).

**Table 2:** Comparison of postoperative parameters between Group 1 and Group 2.

	Group 1(%)(min- max)	Group 2(%)(min- max)	p
Operation Time	195 dk	149 dk	**<0.05**
Amount of Bleeding	320 ml	40 ml	**<0.05**
Bile Tract Complications	3(5.71 %)	1(2.43 %)	>0.05
Clavien Dindo Grade			
Grade 1	12(21.42 %)	6(14.58 %)	**<0.05**
Grade 2	5(8.9 %)	3(7.29 %)	>0.05
Grade 3A	2(3.6 %)	1(2.43 %)	>0.05
Grade 3B, 4, 5	0	0	–
Length of Hospital Stay⁎	5.6(3–28)day	2.2(1−11)day	**<0.05**
Recurrence	0	0	–
Mortality	0	0	–

⁎ After operation.

In Group 2, preoperative imaging showed no pathology in the gallbladder. The average follow-up period was 18 months (min/max: 3–60 months). During this follow-up, 8 patients (19.5 %) developed stones or sludge, and 1 patient (2.4 %) developed a polyp, as detected in postoperative radiological evaluations. Four patients (9.75 %) presented to the emergency department with symptoms of cholecystitis. Four symptomatic patients and 1 patient with a gallbladder polyp (total 5 patients, 12.1 %) underwent elective cholecystectomy. All of these patients' pathology results were consistent with chronic cholecystitis due to cholelithiasis. The patient operated on for a polyp also had a pathology result consistent with chronic cholecystitis due to cholelithiasis, and the polyp in the gallbladder lumen was not pathologically confirmed. Four patients (9.75 %) who had not yet undergone surgery were recommended to have cholecystectomy, but there was no information available in the electronic records confirming that these patients had undergone the procedure (Table 2, Table 3).

**Table 3:** Clinical features related to cholelithiasis in the postoperative follow-up of patients in Group 2.

Group 2	N(%)(min-max)
Median Follow-up Time	18mounth (min-max:3–60)
Cholelytiazis and Polyp	9(21.95 %)
Postoperative Complaints	
Dyspepsia	17(41.45 %)
Biliary Colic	9(21.95 %)
Right Upper Quadrant Pain	6(15.33 %)
Constipation	16(39.02 %)
Cholecystectomy	5(12.9 %)
Type of Cholecystectomy	
Laparascopicaly	2(4.8 %)
Open⁎	3(7.2 %)

⁎ Conversion from laparoscopy.

## Discussion

Surgical treatment of liver hydatid cysts typically involves cystotomy, cavity lavage, and capitonnage, and less frequently, pericystectomy or hepatectomy. There is limited information in the literature regarding the necessity of simultaneous cholecystectomy during these procedures, particularly in conservative surgeries. In this study, the majority of patients who underwent cholecystectomy during hydatid cyst surgery (89.28 %) had pathology results consistent with chronic cholecystitis. On the other hand, 21.95 % of patients who did not undergo cholecystectomy developed gallstones and related symptoms during follow-up, and cholecystectomy was recommended for all these patients. This study is one of the early studies into the necessity of concurrent cholecystectomy during hydatid cyst surgery, proposing that it may be beneficial.

Risk factors for the formation of gallstones include advanced age, female sex, rapid weight gain or loss, obesity, a diet rich in cholesterol and fats, cirrhosis, estrogen use, medications or surgeries affecting gallbladder contractility, and medications, diseases, or surgeries affecting bile absorption [16,17]. It is also known that liver, pancreas, and stomach surgeries can impact the anatomy and function of the bile ducts [10,12,[16], [17], [18]]. In hepatectomies, cholecystectomy is frequently performed. Some studies suggest that the gallbladder can be preserved without increasing the risk of future gallbladder pathology; most of these studies are related to donor left hepatectomies [10,18]. The gallbladder's neural innervation, anatomy, and physiology are generally considered to be disrupted following hepatectomy, leading to the addition of cholecystectomy in these patients [12,19,20]. According to recent publications in Turkey, the incidence of cholelithiasis is 7.6 %. However, in our study, we observed that 21.95 % of patients in Group 2 developed cholelithiasis or gallbladder sludge [21]. This condition may not only be attributed to metabolic predisposition and dietary habits, but also to gallbladder dysfunction related to hydatid cyst surgery. But, there is a lack of studies reporting the development of cholelithiasis and the subsequent need for cholecystectomy following hydatid cyst surgery. Since radical hydatid cyst surgery involves hepatectomy, cholecystectomy is generally performed as well, and in our series, all cases undergoing radical surgery also had cholecystectomy. In contrast, conservative hydatid cyst surgery involves controlled cyst opening and drainage, with the surgical field limited to the cyst itself. In most cases, there is no need to intervene in other organs. Therefore, unlike in radical surgery, it can be assumed that the innervation and anatomy of the gallbladder remain unaffected. However, hydatid cyst surgery can independently cause anatomical and functional disorders in the gallbladder, so the potential for cholelithiasis should not be ignored. Indeed, our study found that some patients who underwent conservative hydatid cyst surgery developed cholelithiasis during follow-up, suggesting the potential necessity of performing cholecystectomy alongside conservative hydatid cyst surgery.

Cholecystectomy is performed not only to prevent postoperative symptoms of cholelithiasis but also to improve exposure during hilar dissection, test for bile leakage from the cyst cavity, and utilize the cystic duct during cholangiography [12,14]. For instance, Yücel et al. [22] reported performing cholecystectomy in 31 % of their patients undergoing surgery for hydatid cysts, primarily to facilitate bile duct exploration in cases where the cyst communicated with the bile ducts. Similarly, Zahairi et al. [23] performed cholecystectomy in cases with gallstones, when bile duct exploration was necessary, and to facilitate access to the cyst. Another study by Yağcı et al. [24] included cholecystectomy and T-tube placement in cases where the common bile duct and extrahepatic bile ducts were obstructed by cyst eggs. Kayaalp et al. [14] routinely tested for bile leakage from the hydatid cyst cavity in all patients by performing cholecystectomy and administering saline through the cystic duct, which they found reduced bile fistulas. These studies indicate that cholecystectomy is performed for secondary reasons in the absence of primary gallbladder pathology. Cholecystectomy is also indicated in cases where there is direct cyst-to-gallbladder fistulization or gallbladder invasion by the cyst, although this is rare [25,26]. A review of the literature indicates no specific mention of whether cholecystectomy was performed when these secondary indications were absent, and there is often no information on symptoms related to cholelithiasis or hospital admissions during follow-up [[27], [28], [29]]. Our study examines also complaints related to cholelithiasis after hydatid cyst operation. In our series, a significant proportion of patients in Group 2 (21.95 %) required cholecystectomy during follow-up, and a large portion of pathology results from Group 1(89.28 %) were reported as consistent with chronic cholecystitis. Considering these findings, performing simultaneous cholecystectomy may be beneficial in preventing future gallbladder-related symptoms and overcoming the challenges of a potential secondary surgery.

The predominant technique employed in hydatid cyst surgery is conservative surgery, regardless of whether it is laparoscopic or open. [4,[30], [31], [32]]. Although radical surgeries such as hepatic resections and pericystectomies reduce recurrence rates (2 %–10 %), they are less frequently performed [25,27,30]. Postoperative follow-up after conservative surgery shows recurrence rates of 5 %–25 % [4,29]. While no recurrences were observed in our series, the average follow-up period of 18 months may be misleading. Additionally, no data were found demonstrating a relationship between simultaneous cholecystectomy and recurrence of hydatid cysts. However, any recurrence of hydatid cyst could increase inflammation in the liver and surrounding tissues, making subsequent cholecystectomy more challenging if gallbladder pathology is present. Furthermore, performing cholecystectomy during a second surgery in the case of recurrence could complicate the third operation. In our series, despite starting laparoscopically, most of the cholecystectomies performed in Group 2 (*n* = 5) were converted to open procedures, with 60 % (*n* = 3) being converted, and hospital stays were prolonged.

When examining the pathological findings of Group 1, chronic cholecystitis was observed in 89 % of the cases, while cholelithiasis and chronic cholecystitis coexisted in 81 % [33]. In our series, the rate of chronic cholecystitis was similar, whereas the coexistence of cholelithiasis and chronic cholecystitis was 28 %, which is lower than reported in the literature. This discrepancy may be attributed to cholecystectomies performed for bile duct exploration and those added to hepatectomy procedures. Interestingly, a significant number of patients without cholelithiasis were also found to have chronic cholecystitis. It is possible that inflammation caused by the hydatid cyst led to the development of chronic cholecystitis and eosinophilic cholecystitis. Although we do not have specific data to explain this mechanism, the presence of chronic cholecystitis in a substantial number of patients without cholelithiasis suggests that it contributed to symptoms, reinforcing the appropriateness of performing cholecystectomy. In Group 1, operation duration, amount of bleeding, and hospital stay length were significantly higher. Additionally, Clavien-Dindo Grade 2–3 A complications and biliary issues were more frequent in Group 1, although the difference was not statistically significant. This observation was considered to be more related to the outcomes of radical resections, such as T-tube placement in cases where hydatid cysts opened into the bile ducts and postoperative bile fistulas, rather than the addition of cholecystectomy to the procedure.

The most significant limitation of this study is its retrospective nature, which naturally leads to potential data deficiencies. Additionally, the non-homogeneous distribution of the groups is another limitation. Although the necessity of cholecystectomy was not evaluated based on a direct comparison of the groups, the higher prevalence of adverse parameters in Group 1, such as prolonged hospital stay, increased blood loss, and higher complication rates, may be attributed to the inclusion of patients undergoing radical surgery and bile duct exploration in this group, ultimately affecting group homogeneity. This research should be supported by prospective and randomized controlled trials. Recurrence after hydatid cyst surgery is an important factor that needs to be evaluated; however, the relatively short follow-up period in our study limited the ability to optimize this assessment. Another limitation is the lack of homogeneity between the groups; in particular, the presence of hydatid cysts opening into the bile ducts, larger size, and multiplicity in patients of Group 1 likely influenced postoperative complication rates.

## Conclusion

Surgical treatment of liver hydatid cysts is generally associated with low morbidity and mortality. Considering that a significant proportion of patients who underwent simultaneous cholecystectomy had chronic cholecystitis in their pathology reports, and that a considerable number of patients who did not undergo cholecystectomy later developed symptoms related to cholelithiasis, it can be suggested that performing simultaneous cholecystectomy during conservative hydatid cyst surgery may be beneficial.

## CRediT authorship contribution statement

**Hüseyin Fahri Martlı:** Writing – review & editing, Writing – original draft, Visualization, Validation, Supervision, Resources, Project administration, Methodology, Investigation, Formal analysis, Data curation, Conceptualization. **Arzu Hazal Aydın:** Writing – review & editing, Writing – original draft, Supervision, Methodology, Investigation, Conceptualization. **Eda Şahingöz:** Writing – review & editing, Writing – original draft, Supervision, Software, Resources, Methodology, Investigation, Formal analysis. **Derviş Duru:** Writing – review & editing, Writing – original draft, Software, Methodology, Investigation, Formal analysis. **Sadettin Er:** Writing – review & editing, Writing – original draft, Validation, Supervision, Software, Conceptualization. **Nesrin Turhan:** Writing – review & editing, Writing – original draft, Methodology, Formal analysis, Data curation, Conceptualization. **Mesut Tez:** Writing – review & editing, Writing – original draft, Resources, Methodology, Funding acquisition, Formal analysis, Data curation, Conceptualization.

## Ethical approval statement

The study received approval from the Ethics Committee of Ankara Bilkent City Hospital, with the assigned approval number E-244514810.

## Funding

No funding.

## Declaration of competing interest

There is no any conflict of interest between authors.

## Data Availability

Data is available. Authors can bring it from hospitals archive.
